# Supplementary material for: The polyketide to fatty acid transition in the evolution of animal lipid metabolism
Source: Nat Commun. 2024 Jan 3;15:236. doi: 10.1038/s41467-023-44497-0 (PMC10764717; doi:10.1038/s41467-023-44497-0)
Supplement: Supplementary file 1 — Supplementary Information [file 41467_2023_44497_MOESM1_ESM.pdf]

Supplementary Information for:

The polyketide to fatty acid transition in the evolution of animal lipid metabolism

Zhenjian Lin, Feng Li, Patrick J Krug and Eric W. Schmidt

This PDF file includes:

Supplementary Figures 1 to 5
Supplementary Table 1
Legends for Supplementary Data 1 to 13

Other supporting materials for this manuscript include the following:

Supplementary Data 1 to 13

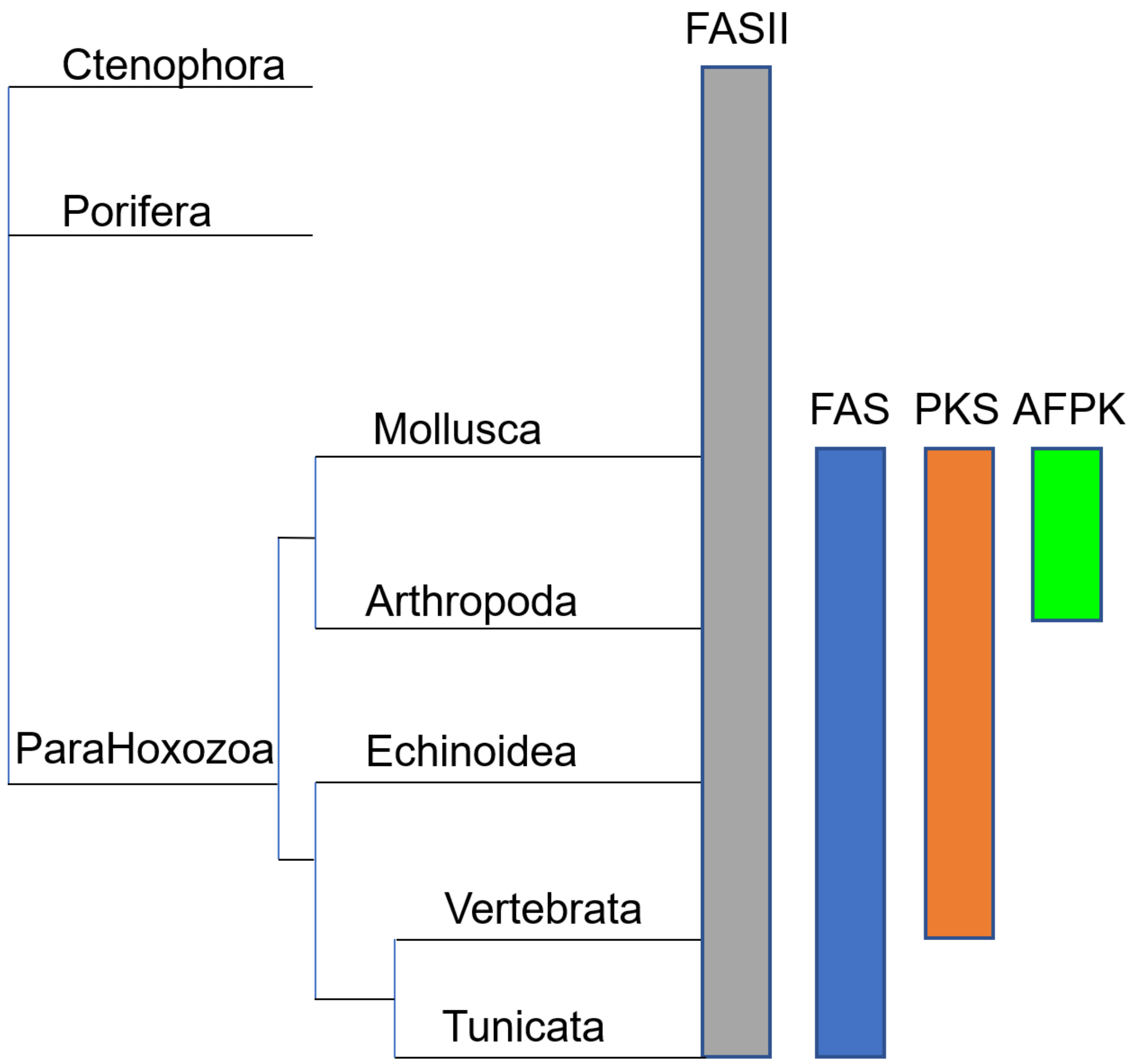

Supplementary Fig. 1. The distribution of the KS-containing genes in different animal phyla. AFPK can be found in Mollusca and Arthropoda.

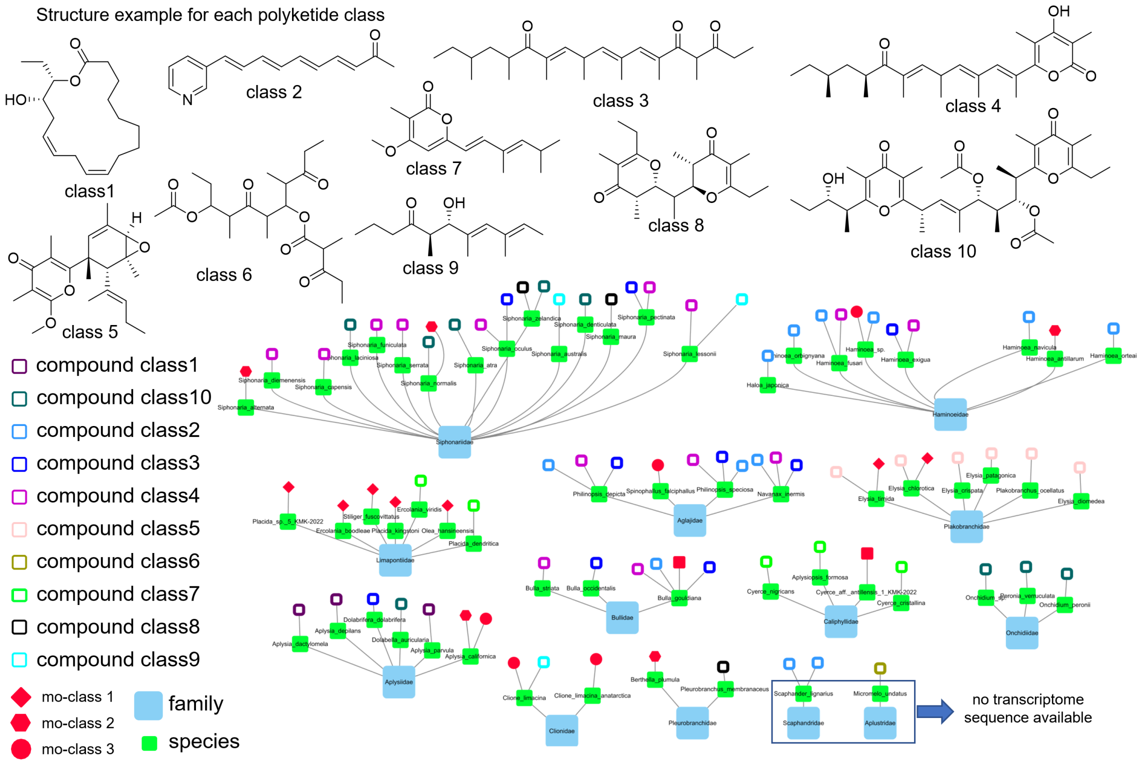

Supplementary Fig. 2. The distribution of the mollusca species that were found to contain polyketide compounds and the species that contain AFPKs. For most of the species that were studied for both chemistry and transcriptome sequences, they fall into the same family. Only Onchidiidae is an exception. There are no transcriptome sequences available for any species in family Scaphandridae or Aplustridae.

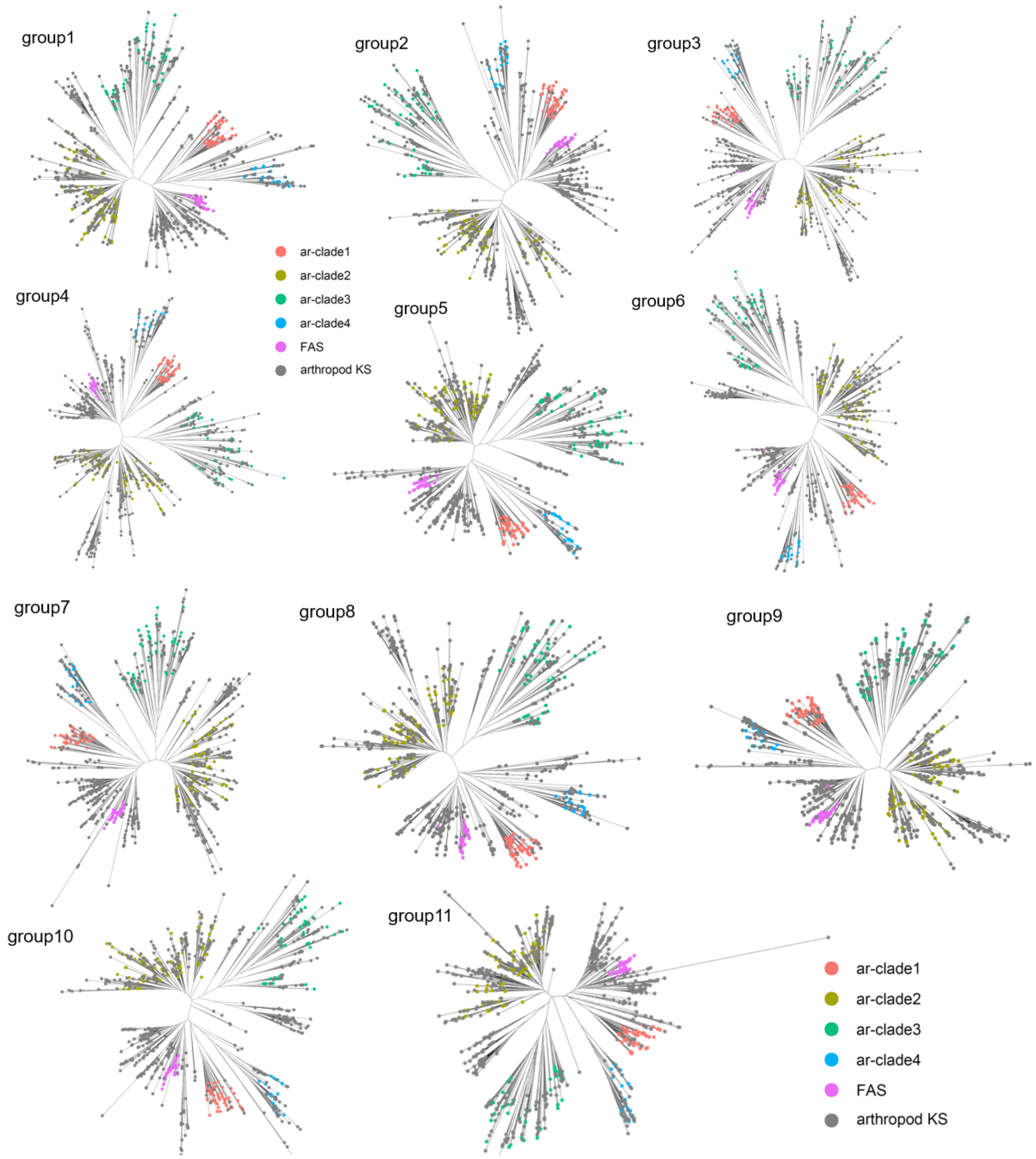

Supplementary Fig. 3. The ML tree analysis of random groups of Arthropoda KSs. The reference sequences were marked using different color in the tips.

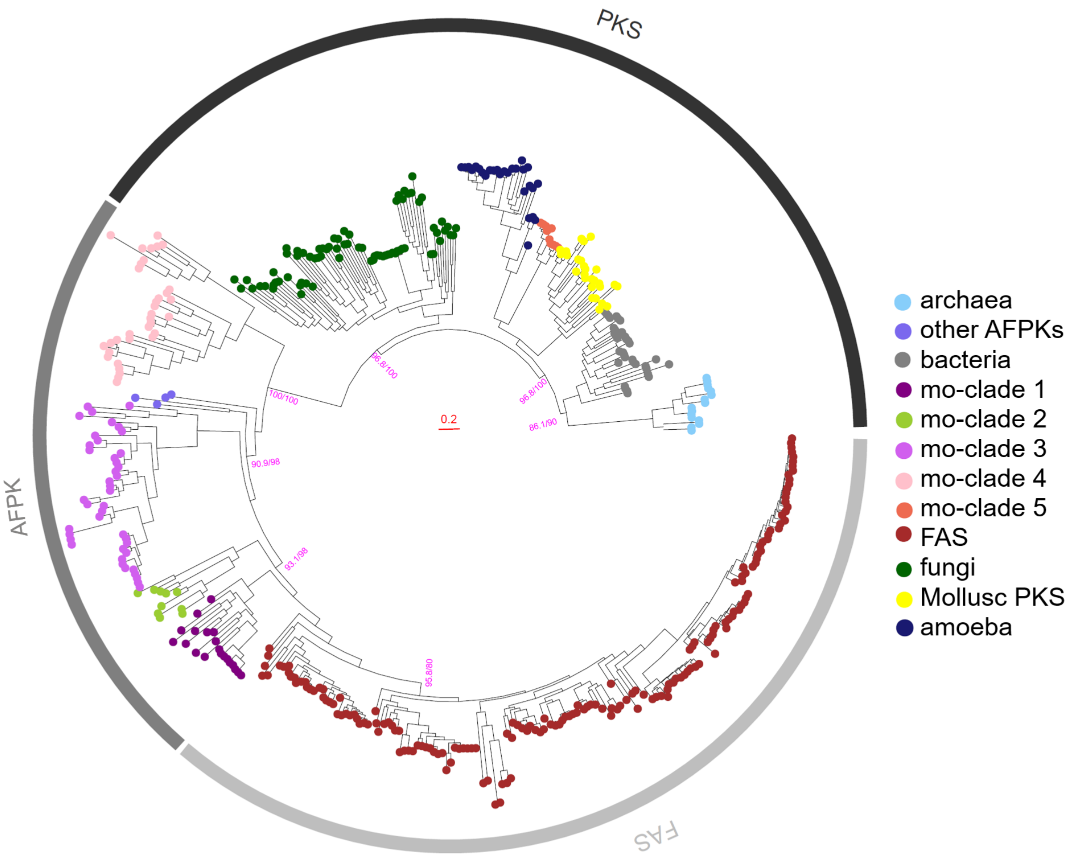

Supplementary Fig. 4. ML tree of selected FAS, mollusca AFPK and PKS KS protein sequences revealed that the animal FAS might derive from an AFPK. Nodes were supported by the Shimodaira-Hasegawa likelihood ratio test and ultrafast bootstrap, given as percent values. Colors of the circles indicate FAS, AFPK, and PKS proteins.

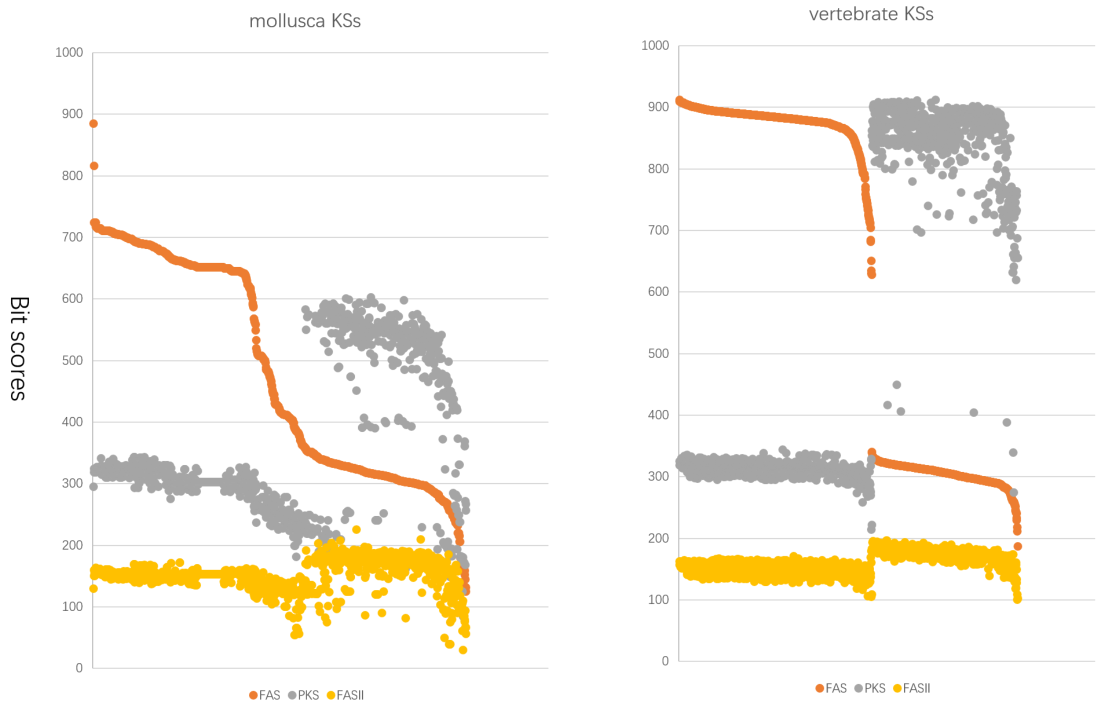

Supplementary Fig. 5. Alignment bit scores of the KS domains to profile HMMs (vertebrate FAS, PKS, and mitochondrial FASII). For the hmm scores in the order FAS, PKS, FASII for EcPKS1 (507.4 263.9 123.1) and (501.0 278.2 133.5), only the FAS HMM scores are lower compare to the values in Figure 2, but these two KSs are still in a discrete region bridging the animal type I FAS and the animal PKSs. FASII sequences were not included in the analysis.

Supplementary Table 1. The number of SRA data sets analyzed in this study.

Phylum	Mollusca	558
Phylum	Porifera	896
Subphylum	Vertebrata	482
Phylum	Arthropoda	4166
Class	Echinoidea	732
Subphylum	Tunicata	232

File Name: Supplementary Data 1

Description: The alignment file in FASTA format for making ANIMAL_KS HMM model used in Figures 2 and 6.

File Name: Supplementary Data 2

Description: The alignment file in FASTA format for making FAS_KS HMM model used in Figures 2 and 6.

File Name: Supplementary Data 3

Description: The alignment file in FASTA format for making FASII_KS HMM model used in Figures 2 and 6.

File Name: Supplementary Data 4

Description: The alignment file in FASTA format for making tree in Figure 3A.

File Name: Supplementary Data 5

Description: The alignment file in FASTA format for making mollusk FAS_KS HMM model used in Figure 3B.

File Name: Supplementary Data 6

Description: The alignment file in FASTA format for making mollusk PKS_KS HMM model used in Figure 3B.

File Name: Supplementary Data 7

Description: The alignment file in FASTA format for making tree in Figure 6B tree1.

File Name: Supplementary Data 8

Description: The alignment file in FASTA format for making tree in Figure 6B tree2.

File Name: Supplementary Data 9

The alignment file in FASTA format for making tree in Figure 7.

File Name: Supplementary Data 10

Description: Lists of SRA accession numbers.

File Name: Supplementary Data 11

Description: GBK_file_for_domain_architecture_visualization in Figure_4A.

File Name: Supplementary Data 12

Description: GBK_file_for_intron_visualization in Figure_4A.

File Name: Supplementary Data 13

Description: The alignment file in FASTA format for making tree in Figure S4.
