# Supplementary material for: The polyketide to fatty acid transition in the evolution of animal lipid metabolism
Source: Nat Commun. 2024 Jan 3;15:236. doi: 10.1038/s41467-023-44497-0 (PMC10764717; doi:10.1038/s41467-023-44497-0)
Supplement: Supplementary file 3 — Description of Additional Supplementary Files [file 41467_2023_44497_MOESM3_ESM.pdf]

Description of Additional Supplementary Files

File Name: Supplementary Data 1
Description: The alignment file in FASTA format for making ANIMAL_KS HMM model used in Figures 2 and 6.

File Name: Supplementary Data 2
Description: The alignment file in FASTA format for making FAS_KS HMM model used in Figures 2 and 6.

File Name: Supplementary Data 3
Description: The alignment file in FASTA format for making FASII_KS HMM model used in Figures 2 and 6.

File Name: Supplementary Data 4
Description: The alignment file in FASTA format for making tree in Figure 3A.

File Name: Supplementary Data 5
Description: The alignment file in FASTA format for making mollusk FAS_KS HMM model used in Figure 3B.

File Name: Supplementary Data 6
Description: The alignment file in FASTA format for making mollusk PKS_KS HMM model used in Figure 3B.

File Name: Supplementary Data 7
Description: The alignment file in FASTA format for making tree in Figure 6B tree1.

File Name: Supplementary Data 8
Description: The alignment file in FASTA format for making tree in Figure 6B tree2.

File Name: Supplementary Data 9
The alignment file in FASTA format for making tree in Figure 7.

File Name: Supplementary Data 10
Description: Lists of SRA accession numbers.

File Name: Supplementary Data 11
Description:
GBK_file_for_domain_architecture_visualization in Figure_4A.

File Name: Supplementary Data 12
Description: GBK_file_for_intron_visualization in Figure_4A.

File Name: Supplementary Data 13
Description: The alignment file in FASTA format for making tree in Figure S4.
