# Supplementary material for: The polyketide to fatty acid transition in the evolution of animal lipid metabolism
Source: Nat Commun. 2024 Jan 3;15:236. doi: 10.1038/s41467-023-44497-0 (PMC10764717; doi:10.1038/s41467-023-44497-0)
Supplement: Supplementary file 17 — Reporting Summary [file 41467_2023_44497_MOESM17_ESM.pdf]

nature portfolio

Corresponding author(s): Eric W. Schmidt

Last updated by author(s): Nov 14, 2023

Reporting Summary

Nature Portfolio wishes to improve the reproducibility of the work that we publish. This form provides structure for consistency and transparency in reporting. For further information on Nature Portfolio policies, see our Editorial Policies and the Editorial Policy Checklist.

Statistics

For all statistical analyses, confirm that the following items are present in the figure legend, table legend, main text, or Methods section.

n/a	Confirmed	
☒	☐	The exact sample size (*n*) for each experimental group/condition, given as a discrete number and unit of measurement
☒	☐	A statement on whether measurements were taken from distinct samples or whether the same sample was measured repeatedly
☒	☐	The statistical test(s) used AND whether they are one- or two-sided<br>*Only common tests should be described solely by name; describe more complex techniques in the Methods section.*
☒	☐	A description of all covariates tested
☒	☐	A description of any assumptions or corrections, such as tests of normality and adjustment for multiple comparisons
☒	☐	A full description of the statistical parameters including central tendency (e.g. means) or other basic estimates (e.g. regression coefficient) AND variation (e.g. standard deviation) or associated estimates of uncertainty (e.g. confidence intervals)
☒	☐	For null hypothesis testing, the test statistic (e.g. *F*, *t*, *r*) with confidence intervals, effect sizes, degrees of freedom and *P* value noted<br>*Give P values as exact values whenever suitable.*
☒	☐	For Bayesian analysis, information on the choice of priors and Markov chain Monte Carlo settings
☒	☐	For hierarchical and complex designs, identification of the appropriate level for tests and full reporting of outcomes
☒	☐	Estimates of effect sizes (e.g. Cohen's *d*, Pearson's *r*), indicating how they were calculated

Our web collection on statistics for biologists contains articles on many of the points above.

Software and code

Policy information about availability of computer code

Data collection	Trimmomatic-0.39, SPAdes-3.15.3-Linux, Prodigal V2.6.3, antiSMASH 5.0, Protein-Protein BLAST 2.13.0+, Autometa 1.2, SDDC v1.0, YAMB 2.0.
Data analysis	T-COFFEE Version_13.45.0.4846264, clipkit 1.3.0, IQ-TREE multicore version 2.2.0.3, ggtree v3.0.4, hmmer3.0, AFPK-finder (https://github.com/linzhenjian/AFPK_finder), ggplot2, ComplexHeatmap 3.17, circlize_0.4.15

For manuscripts utilizing custom algorithms or software that are central to the research but not yet described in published literature, software must be made available to editors and reviewers. We strongly encourage code deposition in a community repository (e.g. GitHub). See the Nature Portfolio guidelines for submitting code & software for further information.

Data

Policy information about availability of data

All manuscripts must include a data availability statement. This statement should provide the following information, where applicable:

- Accession codes, unique identifiers, or web links for publicly available datasets
- A description of any restrictions on data availability
- For clinical datasets or third party data, please ensure that the statement adheres to our policy

The alignment files for HMMs and trees are provided in the Supporting Information. KSs sequences from molluscs, arthropods, and vertebrates used in this study are deposited in figshare: 10.6084/m9.figshare.24066234. Raw sequencing data for the mollusc Siphonaria is available in genbank (SRR22547485 and

SRR22547486). The original data for plotting in figures are provided in Source Data files. The lists of SRA accession numbers that were used in this paper are provided in Supporting Information.

Research involving human participants, their data, or biological material

Policy information about studies with human participants or human data. See also policy information about sex, gender (identity/presentation), and sexual orientation and race, ethnicity and racism.

Reporting on sex and gender	NA
Reporting on race, ethnicity, or other socially relevant groupings	NA
Population characteristics	NA
Recruitment	NA
Ethics oversight	NA

Note that full information on the approval of the study protocol must also be provided in the manuscript.

Field-specific reporting

Please select the one below that is the best fit for your research. If you are not sure, read the appropriate sections before making your selection.

☐ Life sciences ☐ Behavioural & social sciences ☒ Ecological, evolutionary & environmental sciences

For a reference copy of the document with all sections, see nature.com/documents/nr-reporting-summary-flat.pdf

Ecological, evolutionary & environmental sciences study design

All studies must disclose on these points even when the disclosure is negative.

Study description	We sampled sequences from animal phyla to determine the presence of FAS, PKS, and AFPK genes.
Research sample	We included sequencing data from Mollusca (558 specimens), Porifera (896 specimens), Chordata (482 from Vertebrata, 232 from Tunicata), Arthropoda (4,166 specimens), Echinoidea (732 specimens). In addition, we searched the NCBI nr database for animal KS containing proteins, obtained at least 2 (and usually more) specimens from other animal phyla, and obtained representative PKS and FAS sequences from across the tree of life as a comparison data set.
Sampling strategy	All available gastropod SRA datasets available as of January, 2022 were downloaded. For non-gastropod molluscs, the SRA data was sorted in SRA Run Selector by the Bytes column. The top two SRA in byte size were selected for each species and then downloaded. For arthropods and vertebrates, one SRA data set (the top one in the byte size) for each species was downloaded. These sample sizes were sufficient because the purpose of the study was not to statistically evaluate expression in each specimen, but instead to cast a wide net to obtain as many KS-containing sequences as possible from data sets. The sizes were selected to minimize redundancy in processing unique sequences and thus to improve computation time.
Data collection	All KS-containing proteins were collected by HMM search and/or blastp search. the domain boundary were determined by predicted by antiSMASH and InterPro.
Timing and spatial scale	Sequences were obtained from GenBank in January, 2022. All available SRA sequences for the specimens in question present in GenBank were used. Since every deposited sequence was used, it was not necessary to consider timing and spatial scale, as those variables did not apply to an all-inclusive sequence analysis.
Data exclusions	If a transcriptome did not encode a full-length KS sequence, it was excluded from the analysis.
Reproducibility	Each analysis, including phylogenetic tree generation, was performed at least 3 times using slightly different sets of starting sequences to ensure rigorous reproducibility of analysis.<br>We aimed to rigorously obtain all relevant sequences. For example, we searched the SRA database using different levels of taxonomy keywords, such as the phylum name Mollusca, and the class names under Mollusca: Gastropoda, Bivalvia, Cephalopoda, Caudofoveata, Monoplacophora, Polyplacophora and Scaphopoda all the parameters that were used for gene calling, domain prediction, sequencing alignment and trimming, and phylogeny tree making were default or listed in the method section in the paper.<br>All attempts at replication were successful. None of the replicated analyses conflicted with the reported outcomes in the manuscript.
Randomization	NA

Blinding	NA

Did the study involve field work? ☐ Yes ☒ No

Reporting for specific materials, systems and methods

We require information from authors about some types of materials, experimental systems and methods used in many studies. Here, indicate whether each material, system or method listed is relevant to your study. If you are not sure if a list item applies to your research, read the appropriate section before selecting a response.

Materials & experimental systems

n/a	Involved in the study
☒	☐ Antibodies
☒	☐ Eukaryotic cell lines
☒	☐ Palaeontology and archaeology
☐	☒ Animals and other organisms
☒	☐ Clinical data
☒	☐ Dual use research of concern
☒	☐ Plants

Methods

n/a	Involved in the study
☒	☐ ChIP-seq
☒	☐ Flow cytometry
☒	☐ MRI-based neuroimaging

Animals and other research organisms

Policy information about studies involving animals; ARRIVE guidelines recommended for reporting animal research, and Sex and Gender in Research

Laboratory animals	In addition, live specimens of Siphonaria sp. were purchased and shipped from AlgaeBarn.com to the University of Utah in aquarium bags with seawater, inflated with oxygen, obtained in 2021. Animals were sacrificed for sequencing.
Wild animals	No wild animals were used in the study.
Reporting on sex	NA
Field-collected samples	NA
Ethics oversight	Siphonaria sp. is an invertebrate that does not require ethics oversight for study.

Note that full information on the approval of the study protocol must also be provided in the manuscript.
